# Bilateral flows and rates of international migration of scholars for 210 countries for the period 1998-2020

**DOI:** 10.1038/s41597-024-03655-9

**Published:** 2024-07-24

**Authors:** Aliakbar Akbaritabar, Tom Theile, Emilio Zagheni

**Affiliations:** Department of Digital and Computational Demography, Max Planck Institute for Demographic Research, Rostock, 18057 Germany

**Keywords:** Interdisciplinary studies, Sociology, Databases, Society

## Abstract

A lack of comprehensive migration data is a major barrier for understanding the causes and consequences of migration processes, including for specific groups like high-skilled migrants. We leverage large-scale bibliometric data from Scopus and OpenAlex to trace the global movements of scholars. Based on our empirical validations, we develop pre-processing steps and offer best practices for the measurement and identification of migration events. We have prepared a publicly accessible dataset that shows a high level of correlation between the counts of scholars in Scopus and OpenAlex for most countries. Although OpenAlex has more extensive coverage of non-Western countries, the highest correlations with Scopus are observed in Western countries. We share aggregated yearly estimates of international migration rates and of bilateral flows for 210 countries and areas worldwide for the period 1998–2020 and describe the data structure and usage notes. We expect that the publicly shared dataset will enable researchers to further study the causes and the consequences of migration of scholars to forecast the future mobility of academic talent worldwide.

## Background & Summary

Scientists contribute to research and development in the countries where they are working^1,2^. While there is a wealth of literature on brain drain^3^, brain gain^4^, and brain circulation^5,6^, there is still a lack of reliable data on the migration of scientists^7,8^. Here, we seek to address this gap by leveraging two large-scale sources of bibliometric data: i.e., Elsevier’s proprietary Scopus database^9^ and the openly available OpenAlex^10^ database. Building on previous experiences reported in the literature^11–13^ and empirical work by our group^7,8,14–19^, we evaluate different measurements and analytical strategies. We provide best practices on how to re-purpose bibliometric information to prepare data on migration rates and flow estimates for studying the global movements of scholars. In addition to describing the pre-processing steps, presenting illustrative examples of migration measures and trends, and discussing limitations and pitfalls, we share aggregated estimates at the country level for 200 countries and geographical areas from 1998 to 2020^20^ to enable more detailed future analyses of the global circulation of academic talent.

Preparing and providing public access to high-quality data is a well-established tradition in the scientific field of demography^21^, including in its sub-fields dealing with longevity (e.g., Human Mortality Database^22^) and fertility (e.g., Human Fertility Database^23^). Following this tradition, specific national contexts, such as Nordic nations, have exemplary register data on the main life events of their whole population - including births, deaths, marriages and divorces - that can be used to conduct longitudinal research^24^. Another example is the population-scale data on 17 million registered inhabitants of the Netherlands accumulated by Statistics Netherlands^25^.

By contrast, there is a lack of reliable data for research in the sub-field of demography dealing with migration^26,27^. It is difficult to find high-quality data on migration^21,28^. Some projects aimed at harmonizing migration data worldwide, such as the Integrated Public Use Microdata Series (IPUMS)^29^, have shown that records of migration, even between two neighboring countries, can be inconsistent due to differences in the definitions, methods of data collection, and registration and digitization practices used in the respective countries. For example, estimates of the migration rates between Germany and Poland differ depending on which country is reporting them which is due to differences in definitions leading to under- or over-counting of migration events^30^.

One of the usual approaches to remedying this lack of data on migration is to take the differences in the birth, death, and growth rates of populations between two points in time and to consider them as *unexplained factors*, *error terms*, or *implied* or *estimated migration*^31^ which are used in the balancing equation of population change (see pages 2–3 in^32^). This approach is not, however, a measurement of the actual migration events and consequently, the balancing equation of population change has been extended to consider in- and out-migration^32^.

There have been efforts in the literature to retrospectively estimate migration flows from an origin to a destination country by comparing stock data, i.e., the count of the migrant population currently residing in a specific location in a given period, with population data from the country (or region) of origin at the time of the move^33,34^. These estimates are, however, prone to inaccuracies, as migrants may take intermediary steps between exiting the origin country and arriving in the destination country.

This lack of reliable and longitudinal data on migration flows is more pronounced for specific sub-populations, such as high-skilled migrants^7,8^. Since the advent of digitization, different sources of data have been used to provide estimates of migrant populations, including social media data^27,35,36^. A relatively under-explored data source is the metadata of scientific publications accumulated by publishers or large companies, i.e., bibliometric data, which despite its limitations and shortcomings (discussed later), could provide longitudinal *semi-census* information on scholars and their places of work over time^37–39^.

Bibliometric data have proven useful for demographic research^37,38^, especially for research on scholars as a subset of the high-skilled population —see our group’s prior work as examples^7,8,14–19^—. By re-purposing these data and using academic affiliation addresses, it is possible to construct the mobility trajectories of individual scholars^11–15^. We use bibliometric data as a novel source of *digital traces*^37^ and re-purpose them to answer questions regarding high-skilled and, specifically, scholarly migration flows and rates. The geographic scope of our data encompasses all countries and geographical areas worldwide for which suitable data are available in Scopus^9^ and/or OpenAlex^10^.

Here, we introduce some illustrative examples of our group’s prior empirical evaluations using bibliometric data presenting differing definitions for a migration event in diverse national or global contexts, as the type of research that is possible using bibliometric data^37^. Miranda-Gonzalez *et al*.^14^ performed one of the few studies of internal scholarly migration using bibliometric data of all Scopus-published Mexican scholars from 1996-2018 and their mobility between regions of Mexico. They found that most of the scholars did not move, and that the capital, i.e., Mexico City, was the most preferred destination of emigrants. Akbaritabar *et al*.^19^ extended this work to the global level showing the inter-relationships between the two systems of internal and international migration (speculated previously by Skeldon^21^ and King and Skeldon^26^ which was empirically less investigated due to lack of proper data) for the specific case of scholars that was made possible with re-purposing bibliometric data for migration research. This study highlighted the sub-national disparities in academic talent circulation worldwide where some sub-national regions went through an academic depopulation. Zhao *et al*.^15^ investigated the migration of Scopus-published German scholars from 1996 to 2020, and found that fewer female migrant scholars returned to Germany than male migrant scholars. Zhao *et al*.^7^ provided a gender perspective on the migration of scholars worldwide. Specifically, they addressed a gap in the research on the migration of scholars by examining whether male and female scholars were participating equally in transnational mobility, and how these patterns shifted over time. Their results showed that while female researchers continued to be underrepresented among internationally mobile researchers and migrated over shorter distances, this gender gap was narrowing at a faster rate than the gender gap in the population of general active researchers. Subbotin and Aref^17^ investigated the migration of Scopus-published Russian scholars from 1996 to 2020. Their findings indicated that mobile scholars accounted for 5% of all scholars affiliated with Russia, and that in recent years, the so-called *brain drain* from Russia was replaced with a more balanced *brain circulation*. Sanliturk *et al*.^16^ studied the initial changes in the British academic environment after the Brexit referendum. They found evidence that after Brexit, scholars who started their academic careers in the EU countries had a higher probability of leaving the UK, while scholars who started their academic careers in the UK had a higher probability of returning to the UK. The results signaled a compositional change rather than a brain drain in the British academic environment, in the years following the Brexit referendum. Sanliturk *et al*.^8^ looked at whether the migration of scholars worldwide was significantly associated with the economic development of countries (in terms of Gross Domestic Product (GDP) per capita). They found that, on average, emigration propensity initially increased with economic development. However, they observed the opposite pattern for the migration of scholars: i.e., they detected a U-shaped pattern for academics. This means that as GDP increased, the migration of scholars first decreased and then started to increase in rich countries, which might signal the return migration of graduates to their home countries. This is one of the shortcomings of bibliometric data as discussed further below. It is not possible to identify the country of citizenship or nationality of academics. It is only possible to use the country of academic origin, i.e., where one starts their publication activity as the origin location for the first migration event.

Through the studies described above, our group was focused on specific national contexts or applied a global perspective using different measurement strategies and definitions for migration events. In contrast, the data we present in this article covers all countries and areas worldwide that are included in Scopus and/or OpenAlex, and leverage methods we have developed that can be used to conduct comparative studies at a global scale. In addition, this paper presents the best practices adopted after testing different data pre-processing and analysis strategies, such as those we have presented and discussed in the above-mentioned empirical validations.

## Methods

We use a 2023 snapshot of Scopus data and a 2023 snapshot of OpenAlex data. Because many scholars do not publish in every year, the migration data suffer from left- and right-censoring. To avoid this problem, we only analyze the migration data from 1998 to 2020. We have set these limits because our license terms for Scopus data starts from 1996, and we have chosen to use OpenAlex data from the same years to allow for comparisons. The Scopus publications we have included are *Article* and *Review* document types only to ensure that the metadata on affiliation addresses are of the highest quality (see our prior work investigating quality of affiliations^40^). In addition, we have limited the affiliation addresses delivered by Scopus to *author* affiliation addresses in order to exclude publisher addresses and other types of addresses that are less relevant for tracing scholarly migration. For the OpenAlex publications, *journal article* is the only document type we have included, as journal articles make up the largest share of the database’s indexed publications.

### Scientific entity name disambiguation

It is necessary to ensure that the bibliometric data used are of sufficient quality. A lack of proper data or the use of lower-quality metadata can lead to errors in identifying entities (e.g., authors or academic affiliations) —as shown previously by others^41,42^ and us^40^— and migration events^14^. In other words, a failure to properly identify scholars could cause a merger of the mobility trajectories of different individuals. The organizational and academic addresses (i.e., affiliations) need to be correct for the migration event identification to work and to be reliable.

#### Author name

For author name disambiguation, we use identification numbers added to each unique author by Scopus^9^. Authors who do not have a Scopus author ID or are not indicated as *disambiguated* by Scopus are excluded from our validation analysis and consequently excluded from aggregated counts in the shared data^20^. The author_id identifies all publications of a single author in 94.4% of cases (recall) and has a precision rate of 98.1%, which means that the records of two different authors could be merged by mistake under one author_id in only 1.9% of the cases. The precision and recall rates are quoted from Scopus and the study published by Baas *et al*.^9^, which includes more details on the disambiguation process and the Scopus metadata. The quality of Scopus author IDs in comparison to Web of Science (WOS) and CV information is previously evaluated by others^43^. Further detail on our evaluation of identifying migration events using these IDs in comparison to other data is presented in the technical validation section below. For the OpenAlex data, we use the provided author identification numbers; however, since this is a recent initiative, further studies on the quality of these identification numbers are needed.

#### Organization name

Organization names are disambiguated using the research organization registry (ROR) API and the following steps outlined by Akbaritabar^40^. We use the full affiliation string from Scopus to geo-code the organization name to different granular levels. For instance, “Max Planck Institute for Demographic Research (MPIDR), Rostock, Germany” is one affiliation address. However, different authors who use this affiliation in their publication metadata might provide a different set of details when writing it out: e.g., by including or excluding the name of the city or country, or by adding the name of a department or laboratory. Hence, different versions of this address need to be unified under a unique affiliation identification number to reduce the errors that can occur in identifying changes in affiliation addresses, which are used here as proxies for residential address changes, i.e., migration events. See Akbaritabar^40^ for a more detailed description of the methodology used and a comparison of its performance with other organization name disambiguation methods. Below we describe our pre-processing steps including assigning a country for affiliation addresses missing it.

### Pre-processing steps

**Fig. 1:**
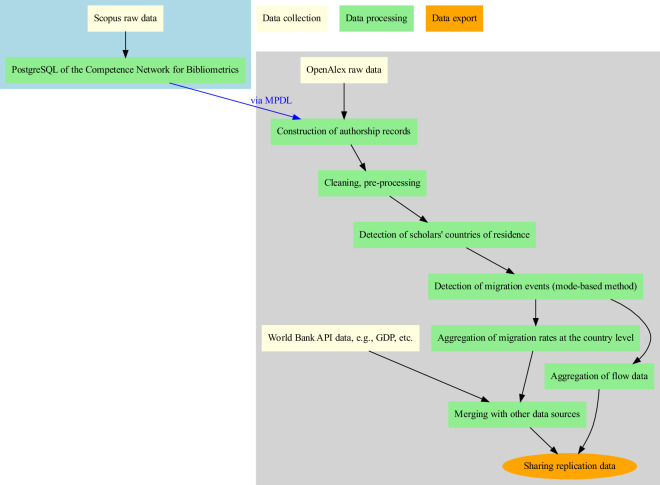
Data collection (light yellow steps), processing (green), and export (orange) pipeline to prepare the migration of scholars dataset^20^, with one step carried out at the Competence Network for Bibliometrics (light blue bounding box) and shared with us via the Max Planck Digital Library (MPDL), and the rest of the steps carried out at the Max Planck Institute for Demographic Research (MPIDR, gray bounding box).

Figure 1 shows the steps involved in the collection, processing, and export of the data, which are described below. The bibliometric data delivered by the database owner, i.e., Scopus, to the German Competence Network for Bibliometrics (Kompetenznetzwerk Bibliometrie, KB)^44^ and through the Max Planck Digital Library (MPDL) to us need to be pre-processed to allow for the identification of migration events. KB prepares a relational database, hosts the Scopus data, and provides us with access through PostgreSQL queries. We obtain the publication data from this database. For OpenAlex^10^, we obtain the publicly available data and process them ourselves at the Max Planck Institute for Demographic Research (MPIDR).

#### Construction of authorship records

For each author, we obtain the list of all publications and process the metadata to assign a date to each author-publication-affiliation triplet. We call this triplet an authorship record^18^. For example, a paper authored by John Doe and Jane Doe, where John Doe has the affiliations “1” and “2” while Jane Doe has only the affiliation “2”, will result in three authorship records as shown in the illustrative example in Table 1. If John has two affiliations in the same year, we reduce the addresses using a mode-based method, which is described further below in the migration event identification section.

**Table 1:** An illustrative example of one publication and its respective authorship records.

Publication Title	Author Full Name	Affiliation	Publication Year
Paper 1 title	John Doe	Affiliation 1, Country 1	2020
Paper 1 title	John Doe	Affiliation 2, Country 2	2020
Paper 1 title	Jane Doe	Affiliation 2, Country 2	2020

#### Country of origin assignment

Bibliometric data do not include the countries of origin of the authors, i.e., their nationalities or citizenships. This is a limitation as we cannot identify a country of origin for migrant scholars. These data only indicate the authors’ countries and addresses of affiliation at the time of publication, which can be used as proxies for their residential addresses, i.e., “country of academic origin”, as we have used in our prior works^7,8,14–19^. Each affiliation includes the address and the country, and in some cases an affiliation_id, which identifies the same institution, even if the name is spelled differently. The address information is available in only 87% of the Scopus records, but the country information is available in 99% of the records^9^. This step entails the harmonization of the country codes and the treatment of missing values. For cases in which the same affiliation address does not have a country assigned to it, we correct for this by filling in the missing country.

In other words, instead of using the country code provided by Scopus or OpenAlex, we train a model to predict the country based on the affiliation text. We then control for the instances in which the code returned by the model is different from the OpenAlex or Scopus country codes. In some cases, OpenAlex mentions a second affiliation in the same text. For instance, in a case in which “Universität Stuttgart; Universidad Barcelona” are indicated as one affiliation, OpenAlex lists a different country than the one returned by the model for this address. We define a high threshold to select the country returned by the model’s prediction (0.92) when it differs from that in OpenAlex. We exclude all records with lower confidence than the set threshold. For example, if for the same author, we have correctly assigned affiliations and incorrect ones, by excluding these problematic ones, we keep the reliable part of the data. This approach enables us to exclude wrongly assigned country codes, and to keep most of those that are correctly assigned.

Previous research has used the author’s family name to find a proxy for the country of origin^45–49^, which could be an avenue for future development to address the shortcoming described above in using country of academic origin instead of citizenship or nationality which is unavailable in bibliometric information.

### Identifying migration events

A migration event is identified based on a change in a scholar’s affiliation addresses. This event can be identified in different ways. One is to include all affiliations per author and any changes in those affiliations as proxies for *travel activity* and *mobility*^15,50^, or to consider only long-term *migration*. We use the definition proposed by the United Nations for international migration that considers a change in usual residential address spanning a minimum of one year^51,52^. We apply this definition consistently to all scholars to detect long-term moves and reduce potential noise caused by short-term mobility and stays. The migration counts and flows are based on the aggregation of all migration events that we can detect using changes in the affiliation addresses of authors.

#### Mode-based method

After the pre-processing of the constructed authorship records, we determine the country of residence for every author and year. Year is the lowest temporal level of analysis for migration events, since the bibliometric data do not provide complete coverage of the month and day of publication. More specifically, we consider all the affiliation countries of an author_id in one year. If there is more than one country, we take the mode of all countries. If there is more than one mode, we check whether one of the modes is the previous country of residence, and take that country as the new country of residence. If that is not the case, we choose one of the mode countries randomly. If our comparison with the previous and next years indicates that the most occurring affiliation, i.e., mode-country, is not changed, we do not record a migration event.

To determine migration events, an algorithm then goes through the years and logs a migration event when the country of academic residence, using mode-based method, changes. We assume a *two-year* preparation time for all publications to cover disciplinary differences in publication delays^53^. If there are gaps in publication years (e.g., if the author is not publishing continuously), we backward fill each publication year for two years and assume that the author’s residence changed two years earlier. If there is enough evidence to do so (i.e., continuous publication activity), we consider the year when the modal affiliation changes as the migration year.

#### Nominator and denominator populations

Once we have detected all migration events, we aggregate them by country and year into emigration and immigration counts, which allows us to calculate other measures such as the net migration rate. To generate measures of exposure (i.e., the denominators for migration rates, or the size of the population of researchers per country and year), we count the number of active scholars for each year and country. The active scholars in a given year include those who have published at least one article or one review during that year.

To deal with missing observations, we assume that an author who has not published in a particular year is still part of the population of active scholars if he or she published one or two years before (two-year backward filling method). Finally, we exclude authors who have *only* one indexed publication during their entire career from the denominator of the scholars’ population. The reason for this exclusion is twofold. First, these scholars might be junior researchers who have just graduated or who have left academia. Since we do not have a live *census* of all academics globally, we cannot consider them as part of the pool of *active scholars*. Furthermore, in each given year, there is a fraction of scholars who enter the pool of active publishers (by having their first publication in the sample) and who exit this pool in the following years^54^. Counting them among the active scholars would over-inflate the population of scholars and cause our measures to be artificially smaller. Second, given that based on the definition of the mode country per year the scholars who have publications in one year only could not have migrated (i.e., contributed to the nominator), it is reasonable to exclude them from the denominator.

#### Bilateral migration flows

Each identified migration event, based on a change in the mode country of affiliation, connects a pair of countries: i.e., an origin country (O) and a destination (D) country. Using these OD pairs and the determined year of migration, we can construct yearly bilateral flows between these countries and origin-destination matrices^33,34^. These matrices are not based on estimates, and they include actual migration events observed in the data as described above. This enables us to identify migration corridors in which a large proportion of scholars move between specific pairs of countries.

### Measures

To evaluate the exposure of populations to migration events, we calculate different measures. We calculate *in-migration* (equation (1)), *out-migration* (equation (2)), and *net migration* counts (equation (3)) and rates (equation (4)) as follows. Rates are standardized using the population of scholars to account for the size differences^14,55^.

1 $$IM{R}_{i,t}=\frac{{I}_{i,t}}{{N}_{i,t}}$$

2 $$EM{R}_{i,t}=\frac{{E}_{i,t}}{{N}_{i,t}}$$

3 $$N{M}_{i,t}={I}_{i,t}-{E}_{i,t}$$

4 $$NM{R}_{i,t}=\frac{{I}_{i,t}-{E}_{i,t}}{{N}_{i,t}}$$

where *i* is the country, *t* is the year, *I*_*i*,*t*_ is the inflow of scholars entering a country, and *E*_*i*,*t*_ is the outflow of scholars exiting that country over the total number of scholars in the country in a given year, i.e., *N*_*i*,*t*_.

## Data Records

The dataset is available at Zenodo^20^ (Link: 10.5281/zenodo.11145735) in form of two tables on global international migration rates and flows (see Table 2). It has been prepared at the country level, and shared publicly under CC-BY license as two CSV files. The release of this dataset is subsequent to a series of prior empirical validation work by our group^7,8,14–19^. We confirm that no individual level raw bibliometric data is shared in the provided dataset^20^. The shared data includes only aggregated and country-level records^20^ that match the data provider’s license terms, i.e., Eslevier’s Scopus. Examples of these datasets are presented here.

**Table 2:** Description of folder structure and files in the produced and publicly accessible dataset^20^.

File name	Description
ReadMe.md	Description file in Markdown format.
scopus_202*_V1_scholarlymigration_country_enriched.csv	Country level yearly dataset on international emigration, immigration, net migration rates and other variables based on Scopus.
scopus_202*_V1_scholarlymigration_countryflows_enriched.csv	Country level yearly “flow” dataset on international emigration, immigration, net migration rates and other variables based on Scopus.
openalex_202*_V1_scholarlymigration_country_enriched.csv	Country level yearly dataset on international emigration, immigration, net migration rates and other variables based on OpenAlex.
openalex_202*_V1_scholarlymigration_countryflows_enriched.csv	Country level yearly “flow” dataset on international emigration, immigration, net migration rates and other variables based on OpenAlex.
01_prepare_enrich_data.py	Source code (Python >=3.9) for downloading World Bank data and merging with Scopus and OpenAlex data.
02_merge_openalex_and_scopus.py	Source code (Python >=3.9) for merging Scopus and OpenAlex data.
03_plotting.py	Source code (Python >=3.9) for plotting Figures 2–6.
04_compare_share_of_mobile_researchers.py	Source code (Python >=3.9) for comparing Scopus, ORCID, and OpenAlex data. Please note, raw data at individual author level is not shared due to license limitations of Scopus. ORCID and OpenAlex data are publicly available.
FIGURES\	Folder with plotted figures in PDF format. Produced by script 03.
data_input\	Folder with input data (aggregated migration events of scholars).
data_processed\	Folder with processed/enriched data. Produced by scripts 01 and 02

For convenience of use, we have included the dataset in both CSV and parquet formats while maintaining the same file names.

Table 3 shows column names and description of them for the *migration rates* data per country-year combination. Each row in the data provided in this table is a country and year combination, with the columns providing information from OpenAlex and Scopus on the count of scholars, the padded population based on the two-year backward filling method described above, and the numbers of incoming and outgoing scholars for this country. This table includes further columns displaying information from the World Bank data and other sources on, for example, the country’s general population, GDP per capita, and income level.

**Table 3:** Description of columns and variables available in the country dataset^20^ table for international migration rates data per country and year combination.

Column name	Description
year	year
countrycode	ISO country code
padded_population_of_researchers	Population of researchers after 2 years backward filling (padding) is applied
number_of_inmigrations	Number of in-migrating scholars
number_of_outmigrations	Number of out-migrating scholars
netmigration	Net migration count
outmigrationrate	Out-migration rate standardized by population of scholars
inmigrationrate	In-migration rate standardized by population of scholars
netmigrationrate	Net migration rate standardized by population of scholars
iso2code	Country ISO 2 letter code
iso3code	Country ISO 3 letter code
countryname	Country name
region	World Bank region
incomelevel	World Bank income level
avg_paddedpop	Average padded population of scholars
gdp_per_capita	Gross Domestic Product (GDP) per capita
population	Population of the country from World Bank

Each row includes one pair of country and year, and the next columns provide information from OpenAlex and Scopus.

Table 4 shows column names and description of them for the *flow* data per country pair and year combination. Each row in the data provided in this table is a pair of two countries, i.e., the origin country (O) and the destination country (D); and the next columns give the count of scholars who have migrated from O to D in the given year based on the Scopus and OpenAlex data.

**Table 4:** Description of columns and variables available in the “flow” dataset^20^ table for international migration flow data per country pair and year combination.

Column name	Description
n_migrations	Number of migrating scholars
year	Year
countrynamefrom	Country name for migration flow’s “origin”
countrynameto	Country name for migration flow’s “destination”
regionfrom	World Bank region from
regionto	World Bank region to
incomelevelfrom	World Bank income level from
incomelevelto	World Bank income level to
gdp_per_capitafrom	GDP per capita from
gdp_per_capitato	GDP per capita to
populationfrom	General population count from
populationto	General population count to
iso3codefrom	ISO 3 letter code from
iso3codeto	ISO 3 letter code to
paddedpopfrom	Padded population of scholars from
paddedpopto	Padded population of scholars to

Each row includes a pair of source- and destination-country and year, and the next columns provide information from OpenAlex and Scopus.

## Technical Validation

We have carried out different validation steps on the described methods presented elsewhere^7,8,14–19^. As an example, we control for the effect of different backward and forward padding settings for publication years (e.g., for the years when a scholar does not publish) and its impact on the migration counts and rates. In addition, we control for the quality of the bibliometric metadata, and consider limits on the subset of the data with sufficient quality to be included in the shared data^20^. This entails excluding specific starting and ending years, affiliation addresses that are not for authors (e.g., for publishing houses), and document types to ensure the reliability of the shared data (as described above).

Here, we present 1) examples of illustrative results that can be obtained from the constructed dataset, and 2) results of our comparison and validation of the size of the population of scholars and the net migration rates based on Scopus and OpenAlex. Furthermore, we present results of individual-level comparison between migration events identified using Scopus data and employment histories self-reported by scholars on Open Researcher and Contributor ID (ORCID) public profiles and country level yearly correlations between these three databases to further ensure the reliability of the shared data^20^ for migration research.

**Fig. 2:**
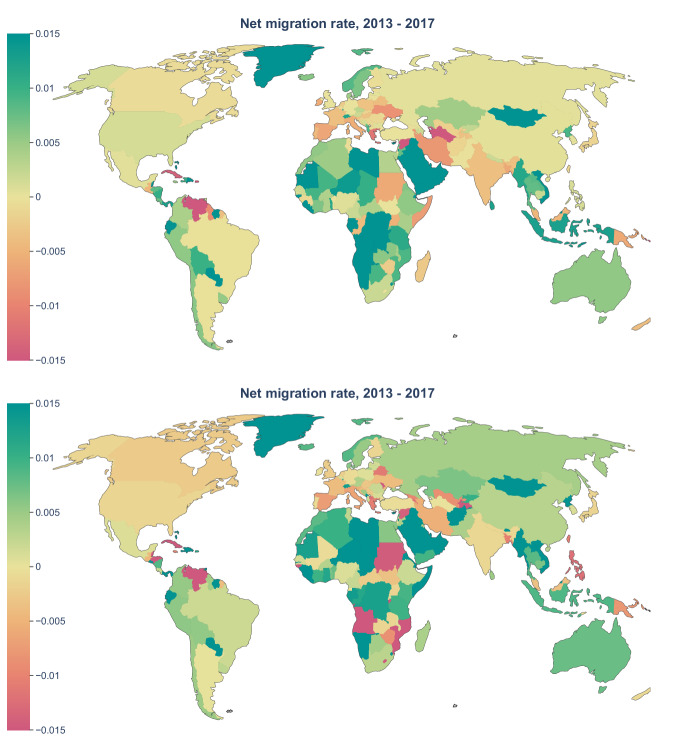
Net migration rates of scholars worldwide from 2013–2017 based on Scopus (top) and OpenAlex (bottom), shown as the rate per 1,000 scholars.

Figure 2 shows the international net migration rates (NMR) worldwide based on Scopus (top) and OpenAlex (bottom) per 1,000 scholars. It shows a consistent pattern for most countries. In some exceptional countries, the NMR calculated using Scopus and OpenAlex differ. For example, in the cases of Canada, Guyana, Costa Rica, Honduras, Bolivia, Russia, China, India, Iran, Turkmenistan, Sudan, Angola, Mozambique, and Philippines, the colors are different in the map on the top than in the map on the bottom.

**Fig. 3:**
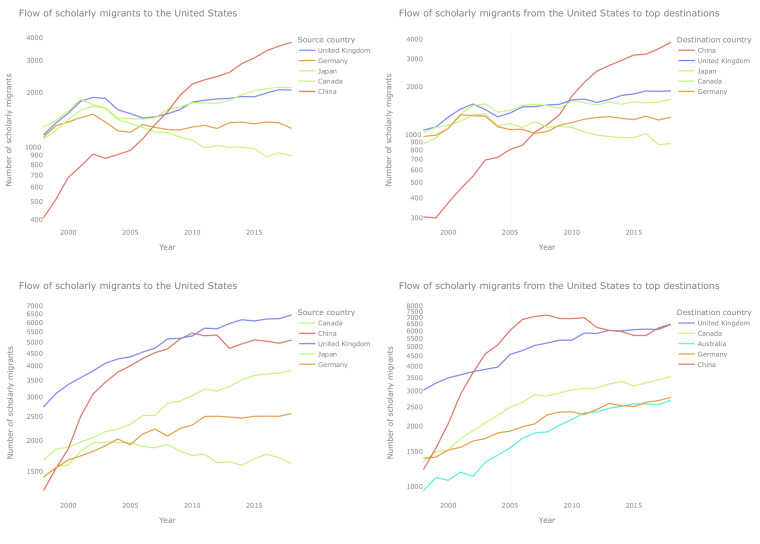
Temporal changes in the migration flows of scholars *to* (left) and *from* (right) the United States based on Scopus (top) and OpenAlex (bottom). The figure is limited to the 5 origin and destination countries with the highest flows. Flows are presented as the actual counts of scholars sent or received, and the 2018 counts can be seen in Fig. 4 as printed labels in cells.

Figure 3 shows the example of the United States and the temporal trend of flows of scholars arriving in the US from other countries (left) and leaving the US to go to other countries (right) based on Scopus (top) and OpenAlex (bottom). The magnitude of the flows based on OpenAlex is much larger than that based on Scopus, and although we have limited the publications in both databases to articles and reviews, this could indicate that the broader coverage of publications in OpenAlex could help us identify some under-explored scholarly migration corridors worldwide. Nevertheless, as was described in the methods section, the quality of the recently introduced author name disambiguation and identifiers in OpenAlex requires further evaluation in future research.

**Fig. 4:**
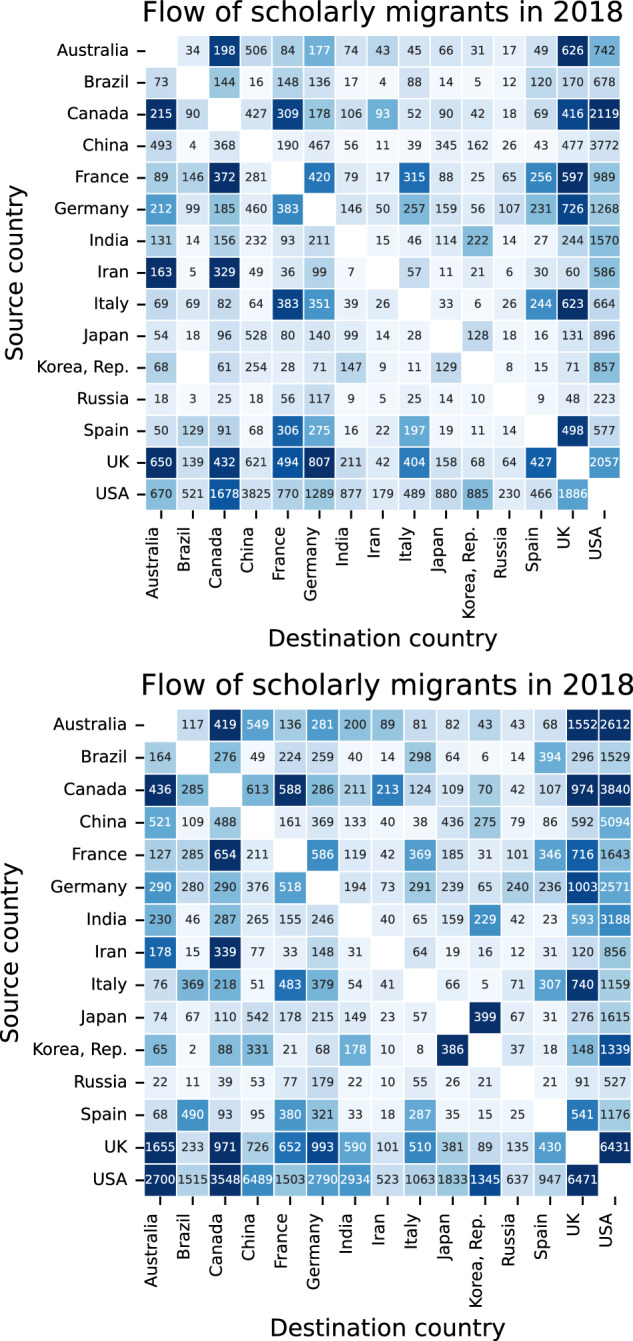
Bilateral flows of scholarly migration between the 15 pairs of countries with the highest exchanges based on Scopus (top) and OpenAlex (bottom). The numbers printed in cells are the actual count of scholars who moved from the source country (Y-axis) to the destination country (X-axis). The colors are based on the normalized flow of migrants. Normalization is done by dividing the total flow of scholars between each country pair by the total outflow from the source country times the total inflow to the destination country.

Figure 4 shows the 15 country pairs with the highest bilateral flows of scholars where the origin (Y-axis) and destination (X-axis) pairs based on Scopus (top) and OpenAlex (bottom) are presented. While in most of these country pairs the colors that are normalized based on the size of the population of scholars are consistent, the printed labels inside the cells that show the actual count of scholars have larger magnitudes in OpenAlex (bottom).

**Fig. 5:**
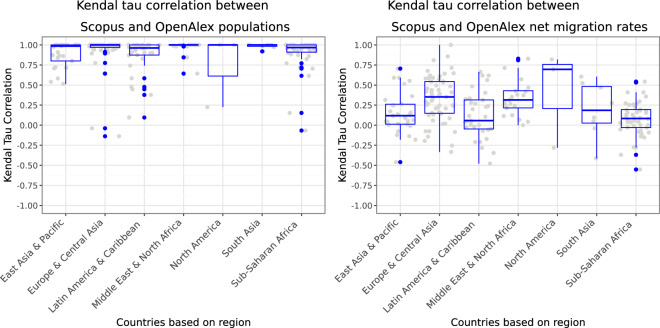
Kendal tau correlation between population (left) and net migration rates (right) from 1998–2018 based on Scopus and OpenAlex divided over different continental regions (X-axis). Each gray dot is one country’s correlation measure, and a jitter is added to the X-axis positioning of dots to reduce their overlap without substantive meaning. The blue boxplots and dots show the trend and the median (thick line) of the same data.

Figure 5 shows the correlation between the population of scholars (left) and the net migration rates (right) across the continental regions. It is clear that while the populations of scholars in the two databases correlate to a high degree over years with a median correlation close to 1, the net migration rates fluctuate to a much higher degree. This could signal large differences in the coverage of individual migration trajectories in these two databases, or it could stem from low net migration rates, which may fluctuate due to small differences in measurement. It is, however, unlikely to stem from population counts, as population counts are larger, and small changes do not cause them to fluctuate.

### Validation with ORCID data

The ORCID is a non-profit organization that provides a registry for unique researcher IDs. Its web service also allows scholars to input CV data and publications into the ORCID database. The data is open and available free of charge as XML data dump.

We downloaded the most recent ORCID data dump from October 2023 and extracted all entries with associated CV information which is employment history self-reported by scholars. We inferred migration events from the changes in the country of employment of individual researchers.

As a further evaluation of our migration event identification method and counts and rates provided in the shared data^20^, we matched individual-level data from Scopus with individual-level data from public ORCID employment histories reported by scholars to provide a validation of our approach consisting the following steps and results:

1. We downloaded the yearly ORCID XML database dump of October 2023 (From: “10.23640/07243.24204912.v1”).
2. We used Python to read through the whole data dump and extract the employment history of individual scholars. We extracted the ORCID-ID, the start year, the end year, the employment organisation, the country of employment and the name of the scholar.
3. We used this extracted information to infer migration events (a migration event happens if the country of employment changes).
4. Scopus also includes ORCID-IDs for a subset of scholars. We extracted all scholars and authorship records with ORCID-IDs from Scopus and also inferred migration events from this data.
5. We merged these two datasets using the ORCID IDs.
6. We compared the migration events based on the country pairs that one has moved between them and the count of migration events identified per scholar from the two different data sources.

Our matching results between Scopus and ORCID at the individual-level indicated the following:

1. The total number of matched ORCID IDs with at least one international migration: 33,695
2. The number of ORCID IDs, where Scopus has a higher count of migration events than ORCID employment history: 3,135 (9.30%)
3. The number of ORCID IDs, where ORCID has a higher count of migration events than Scopus: 13,193 (39.15%)
4. The number of ORCID IDs with the same number of migration events identified using Scopus and ORCID: 17,367 (51.54%)
5. The number of ORCID IDs with exactly the same migration events occurred between the same pair of countries: 11,703 (34.73%)

To summarize the matching results described above, we were able to find 33,695 authors with international migration events in both Scopus and ORCID. For more than 90% of these distinct IDs, scholars had the same or a lower number of migration events identified using Scopus data. This further confirms that our mode-based method and use of publication data to identify international migration events are conservative and less prone to noise. We think the higher count of migration events in ORCID is expected since for many junior scholars the profile and employment history include “PhD student” or “PostDoc” or similar titles which would not be recorded in Scopus affiliation addresses. In addition, creating a public free ORCID profile is less costly, time- and energy-consuming than publishing in a Scopus-indexed journal which further explains the higher count of migration events found using ORCID.

**Fig. 6:**
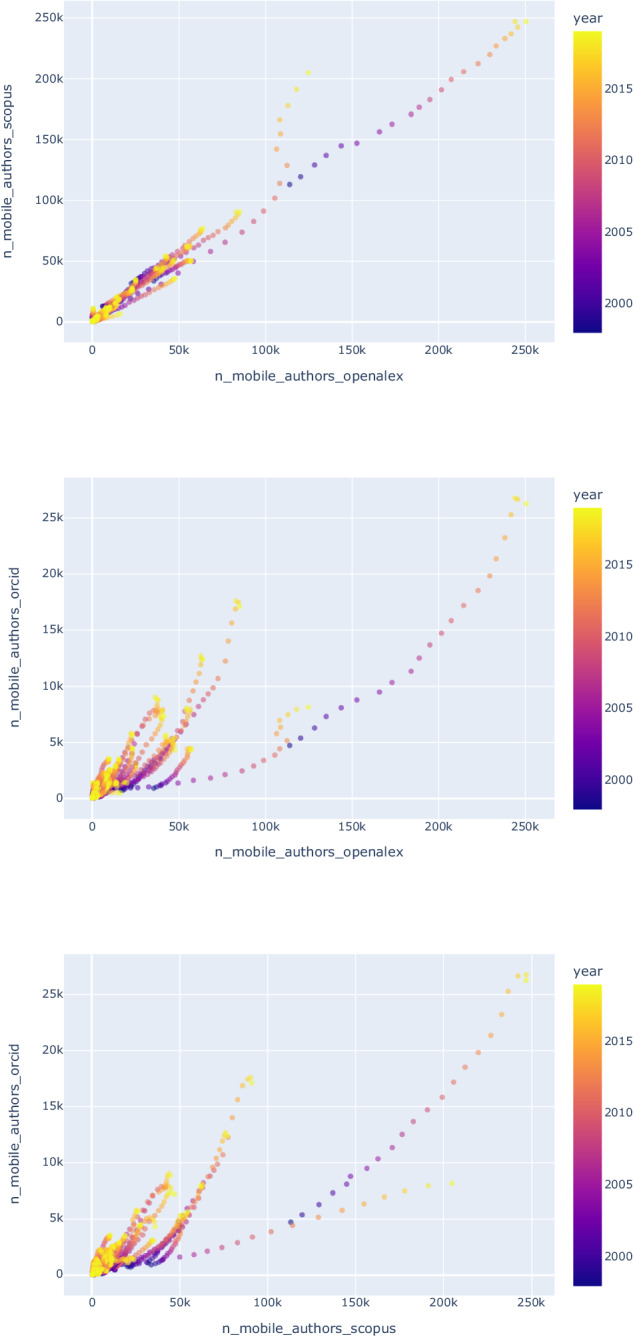
Comparison of the yearly population of mobile scholars per country between OpenAlex and Scopus (top), OpenAlex and ORCID (middle) and Scopus and OpenAlex (bottom).

As a further evaluation of the overall coverage of these three databases, i.e., Scopus, OpenAlex, and ORCID, based on the count of population of scholars, the count, and the share of mobile scholars per year and country, we carried out additional pairwise correlations using Kendall’s Tau. Table 5 and Fig. 6 show the results of these correlations and the yearly counts per country are available in our publicly shared dataset^20^. Overall, the table shows a 0.87, 0.66, and 0.63 correlation between the yearly count of scholars per country, and a 0.86, 0.67, and 0.64 for the count of mobile scholars. In the case of share of mobile scholars per country, correlations are less strong, i.e., 0.52, 0.43, and 0.44. We need to emphasize that we observed higher correlations between Scopus and OpenAlex that was due to the more similar nature of these two databases which are based on publications and affiliation addresses and a consistent change in mode affiliation addresses are used as a measure of migration. Since the nature of ORCID data, which is employment histories reported by scholars, is different from those publication-based databases, and we used it here to further validate our dataset^20^, hence, to have a better baseline of comparison, we used the count of countries throughout a scholar’s career to identify scholars who have been affiliated (for Scopus and OpenAlex) or employed (for ORCID) in at least two or more different countries. Overall, we emphasize that these databases have their limitations, however, to a high degree, they show correlated results per country and year pair despite the lower coverage of ORCID.

**Table 5:** Kendall’s Tau correlation coefficients for the number of scholars in OpenAlex, Scopus, and ORCID by country and year.

Correlation between the yearly population of scholars			
	OpenAlex	Scopus	ORCID
OpenAlex	1.00	0.87	0.66
Scopus	0.87	1.00	0.63
ORCID	0.66	0.63	1.00
**Correlation between the yearly population of mobile scholars**			
OpenAlex	1.00	0.86	0.67
Scopus	0.86	1.00	0.64
ORCID	0.67	0.64	1.00
**Correlation between the yearly shares of mobile scholars**			
OpenAlex	1.00	0.52	0.43
Scopus	0.52	1.00	0.44
ORCID	0.43	0.44	1.00

The first set of three rows show the total number of scholars per country per year in correlation with the database indicated in the column. The second set of three rows show the count of mobile scholars per country per year. The third set of three rows show the correlations between the shares of mobile scholars relative to the country’s population of scholars.

## Usage Notes

Please note that in our joint operation on the Scopus and OpenAlex data and in order to be inclusive, we keep all country-year pairs for which one of these databases has counts. By contrast, in our visualizations, we exclude the rows in which one of the databases does not have measurements for a country-year pair. In using the shared dataset^20^, please consider filtering the rows according to your goals and research question.

## Data Availability

All scripts to replicate the presented validation analysis and figures are publicly accessible under Affero General Public License (AGPL)^20^ alongside the aggregated datasets based on Scopus and OpenAlex on Zenodo at 10.5281/zenodo.11145735 and this ref. ^20^.
